# Effect of AG1^®^ supplementation on nutritional adequacy and gut microbial composition in trained adults

**DOI:** 10.3389/fnut.2026.1783951

**Published:** 2026-03-31

**Authors:** Adam M. Gonzalez, Jeremy R. Townsend, Philip A. Sapp, Caitlyn Edwards, Trevor O. Kirby, Justin Wright, Regina Lamendella, Ralph Esposito

**Affiliations:** 1 Department of Allied Health and Kinesiology, Hofstra University, Hempstead, NY, United States; 2 Research, Nutrition, and Innovation, AG1, Carson City, NV, United States; 3 Health & Human Performance, Concordia University Chicago, River Forest, IL, United States; 4 Department of Nutritional Sciences, The Pennsylvania State University, University Park, PA, United States; 5 Wright Labs, Huntingdon, PA, United States; 6 Department of Nutrition, Food Studies and Public Health, New York University-Steinhardt, New York, NY, United States

**Keywords:** gut health, microbiome, nutrient gaps, prebiotics, probiotics, vitamins

## Abstract

**Background:**

Dietary supplements that combine vitamins, minerals, phytonutrients, prebiotics, and probiotics have gained popularity among health-oriented consumers seeking convenient ways to fill nutritional gaps and support gut health.

**Methods:**

This randomized, double-blind, placebo-controlled crossover study examined the effects of two weeks of the nutritional supplement (AG1^®^) on gut microbial composition, nutritional adequacy, and tolerability. Twenty resistance-trained men (*n* = 10; 26.4 ± 6.5 y) and women (*n* = 10; 26.9 ± 5.3 y) supplemented daily with either AG1^®^ or placebo (PL) for 14 days. Following a 2-week washout, participants crossed over to the other condition. Participants provided stool samples for gut microbial composition analysis, completed the Digestion-associated Quality of Life Questionnaire (DQLQ), and completed a 24-h dietary intake assessment at the beginning and end of each 14-day supplementation period. Outcomes were analyzed using repeated-measures and multivariate statistical approaches for dietary intake, gut microbiota, metabolomics, and questionnaire data.

**Results:**

AG1^®^ did not produce large, global shifts in microbial alpha or beta diversity, supplementation was associated with selective enrichment of key bacterial taxa commonly linked to gut health, including *Lactiplantibacillus plantarum, Lacticaseibacillus rhamnosus*, and *Bifidobacterium animalis*. AG1^®^ supplementation significantly improved nutritional adequacy by increasing the total number of micronutrient Estimated Average Requirements (EARs) met compared to placebo (2.8; *p* = 0.0011), with no significant differences in digestive quality of life between groups (*p* = 0.777). Vitamins A, C, and E were the most common nutrient gaps filled by AG1 supplementation.

**Conclusions:**

Two weeks of AG1^®^ supplementation improved micronutrient adequacy in healthy resistance-trained adults by reducing nutrient gaps. Supplementation also selectively enriched key beneficial gut microbial taxa and putative microbial functional without inducing major disruptions to overall community structure. Importantly, AG1^®^ was well tolerated and did not negatively impact digestion-associated quality of life.

**Clinical trial registration:**

ClinicalTrials.gov, identifier: NCT06521424.

## 1 Introduction

The human gut microbiome is comprised of the trillions of microorganisms, their genes, and their metabolic products, which reside in the gastrointestinal tract and it plays a central role in maintaining overall health and well-being (1). Variations in microbiome composition and function have been linked to a range of outcomes including digestive symptom severity, systemic metabolic effects, immunological modulation, and brain health (2–4). Diet, micronutrient intake, and bioactive compounds such as prebiotics as well as probiotics contribute uniquely to shaping the gut microbiome and supporting host physiology (5–7). Many gut microbes rely on vitamins and minerals to fuel growth and carry out essential metabolic processes. Accordingly, micronutrients such as B-complex vitamins, vitamins C, D, and E, as well as minerals including calcium, iron, zinc, magnesium, and phosphorus, can influence microbial composition and activity (8, 9). Prebiotics are substrates that are selectively utilized by host microorganisms conferring a health benefit (10). They promote the generation of short-chain fatty acids (SCFAs), which support gut health by contributing to epithelial repair, enhancing resistance to gastrointestinal pathogens, and helping maintain immune balance and intestinal homeostasis (9). Probiotics, defined as live microorganisms that provide health benefits when consumed in adequate amounts, can also modulate the gut environment by enhancing populations of beneficial bacteria and yeast, strengthening the mucosal barrier, limiting pathogen adherence, and interacting with immune cells (11). When combined, prebiotics and probiotics function synergistically as synbiotics, with the prebiotic component supporting probiotic survival and activity, thereby amplifying their beneficial effects on gut microbial balance and overall health (6).

Dietary supplements that combine vitamins, minerals, phytonutrients, prebiotics, and probiotics have gained popularity among health-oriented consumers seeking convenient ways to fill nutritional gaps and support gut health. Although not a substitute for whole foods, such supplements can provide concentrated sources of micronutrients, phytonutrients, and microbiome-targeted compounds that help address dietary inadequacies, even among active individuals who strive to maintain a well-rounded and healthy diet (12). Supplementation strategies that deliver a blend of nutritional components may help promote adequate intake of key micronutrients, close nutrient gaps, improve overall diet quality, and support gut microbial function. In particular, formulations that pair micronutrients with prebiotics and probiotics may further enrich beneficial microbial taxa, enhance SCFA production, and support microbial balance, contributing to improvements in gut and systemic health beyond just prebiotics and probiotics alone (6, 7, 11, 13).

AG1^®^ is a commercially available nutritional supplement formulated to deliver a complex blend of micronutrients, prebiotics, probiotics, phytonutrients, and adaptogenic botanicals. Key constituents include essential vitamins and minerals, fermentable fibers, lactic acid bacterial probiotic strains, and plant-based ingredients like whole fruit and vegetable powders and adaptogens. Prior investigations have explored AG1′s effects *in vitro* using simulated gut ecosystems demonstrating that an acute dose can shift microbial taxa and functional pathways (14–17). AG1^®^ has also demonstrated greater digestibility than traditional multivitamin tablets in an *in vitro* gastrointestinal model, which may enhance its bioaccessibility and bioavailability and potentially reduce gastrointestinal side effects often associated with traditional multivitamin formulations (18). Most recently, La Monica and colleagues (19) showed that AG1^®^ supplementation over 4 weeks in healthy adults enriched probiotic taxa, including *Lactobacillus acidophilus* and *Bifidobacterium bifidum*, and modulation of microbial functional pathways without adverse effects on gastrointestinal tolerability or clinical safety markers.

The effects of multi-ingredient nutritional supplements on gut microbiome composition and dietary adequacy in physically trained populations remain underexplored. To expand upon the potential role of novel nutrition supplements in supporting the gut microbiome composition, this current study assessed how 2 weeks of daily AG1^®^ supplementation influences gut microbiome composition and function, nutritional adequacy, and digestive quality of life compared to placebo in resistance trained adults. It was hypothesized that AG1^®^ would favorably modulate the gut microbiome and address nutritional inadequacies without altering measures of digestive quality of life.

## 2 Materials and methods

### 2.1 Experimental design

**Figure 1:**
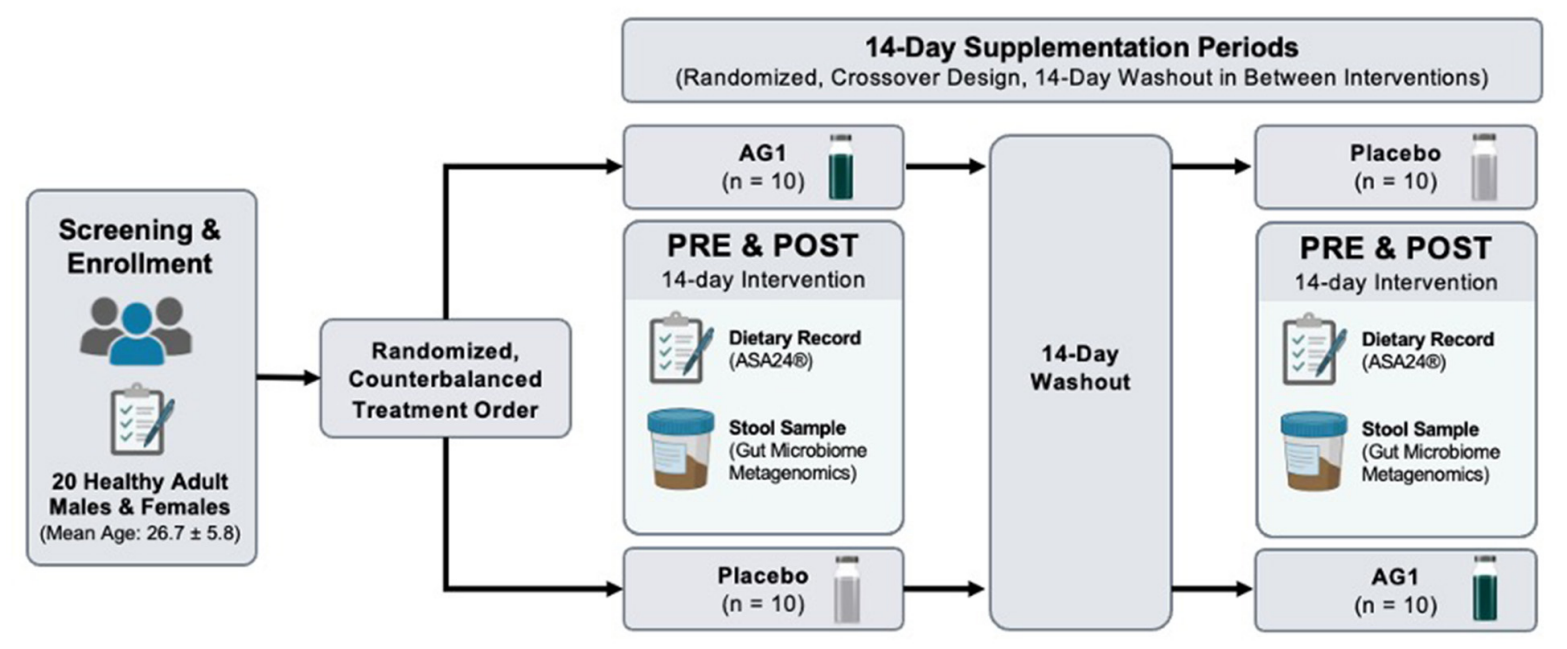
Randomized crossover study design.

This was a randomized, double-blind, placebo-controlled crossover designed study. The primary outcome was to evaluate nutritional adequacy in trained adults following supplementation. The secondary outcomes included the assessment of changes in gut microbial composition and function and the assessment of the tolerability of consuming AG1^®^. Participants supplemented daily with either AG1^®^ or placebo (PL) for 14 days. Following a 2-week washout, participants crossed over to the other condition following International Scientific Association for Probiotics and Prebiotics (ISAPP) guiding principles for synbiotic research (20). Participants were instructed to collect a stool sample at home before starting each 14-day supplement regimen and again within 1 day of completing each regimen following the 14 days. Participants returned the stool sample to the lab to be frozen and used for subsequent analysis. Participants completed the Digestion-associated Quality of Life Questionnaire (DQLQ) and a 24-h dietary intake assessment at the start and end of each 14-day supplementation period. The study design is depicted in Figure 1. Participants were asked to follow their normal diet and activity patterns throughout their participation in the study.

### 2.2 Participants

Twenty resistance-trained men (*n* = 10; 26.4 ± 6.5 y; 174.5 ± 9.8 cm; 85.8 ± 9.6 kg; 10.3 ± 7.9 years of resistance training experience) and women (*n* = 10; 26.9 ± 5.3 y; 163.8 ± 8.7 cm; 66.1 ± 13.0 kg; 9.1 ± 5.4 years of resistance training experience) participated in this study. Inclusion criteria required participants to have >1 year of resistance training experience (minimum of 2 days per week of resistance training), be free of any chronic illness, be free of any medication use, and be free of any prebiotic, probiotic, or fiber supplementation. Participants were also excluded if they had a known chronic condition or a known allergy to any of the ingredients in the supplement or placebo. All procedures were approved by the University Institutional Review Board prior to enrollment, and all participants provided written informed consent. The trial was registered on clinicaltrials.gov (NCT06521424).

### 2.3 Supplementation

All supplements (AG1^®^ and placebo) were prepared and packaged in coded generic single sachets for consumption throughout the study protocol. These white sachets arrived at the laboratory with a printed code to blind both the researchers and participants. Participants were instructed to mix the powder with 500 ml of water for daily ingestion. The nutritional information and ingredients for AG1^®^ (Athletic Greens International, Carson City, NV, USA) are provided in Supplementary Figure 1. AG1^®^ is certified to have undergone evaluation and verification via NSF testing (Ann Arbor, MI, USA) to ensure the product meets strict quality, purity, safety, and label accuracy standards. PL contained 13 grams of maltodextrin and was flavored and colored to match AG1 flavoring. Participants were randomly assigned to treatment conditions using block randomization with a fixed block size of four to ensure balanced group allocation throughout the enrollment process. To assist with compliance, participants agreed to a messaging system to provide reminders to consume supplements and collect samples. Verbal confirmation of daily supplement intake was obtained from participants.

### 2.4 Dietary intake assessment

Participants were instructed to complete non-random 24-h dietary recalls to record all food and beverage intake at the beginning and end of each intervention period using the electronic Automated Self-Administered 24-h Dietary Assessment Tool (ASA24^®^, National Institutes of Health, Bethesda, MD, USA) (21). ASA24^®^ was administered per the recommendations by the National Cancer Institute (NCI) Dietary Assessment Primer. One baseline recall was completed during the first week (days 1–7) and one endpoint recall was completed during the second week (days 8–14) of each 14-day supplementation period totaling four dietary recalls per participant. Nutrient data (e.g., calories, fat, vitamin A, zinc) were extracted from the ASA24^®^ analytical output files. Macro- and micronutrient values from AG1^®^ and PL were retroactively added to each respective endpoint recall as individuals were blinded to their condition and these items were not available in the ASA24^®^ nutrient databases. The dietary data were reviewed and cleaned according to the National Cancer Institute (NCI) reviewing and cleaning guidelines (22).

### 2.5 Nutrient gaps analysis

Nutrient gaps were defined as the difference between an individual's reported dietary intake and established dietary targets [the Estimated Average Requirements (EARs) for specific nutrients]. More specifically, the EAR cut-point method was employed to determine the proportion of participants with intakes below the EAR (23). Sixteen micronutrients were included in the assessment based on the following criteria: micronutrients were present in the study product, could be measured by ASA24^®^, and had an EAR. The combined nutrient gap score was determined by adding the nutrient intakes that met the EAR, with each micronutrient meeting the EAR counting as 1, for a total maximum score of 16. Nutrient values from AG1^®^ or placebo were added to the respective endpoint diets, and a nutrient gap score was computed to determine the impact of each treatment.

### 2.6 Stool collection

Stool collection kits were provided to each participant prior to each 14-day supplementation period. Each stool kit supplied a collection tube with an integrated spoon and DNA stabilizer (DNA/RNA Shield Fecal Collection Kit, Zymo Research), a collection tube with an integrated spoon and metabolite stabilize (OMNImet GUT ME-200, DNA Genotek), a clear sample bag, and a bubbled sealed envelope along with detailed instructions for collection. Participants were instructed to collect fecal samples before each 14-day supplement intervention and within 1 day following the intervention. Stool kits were returned to the laboratory and stored at −80 °C until shipped to a third-party laboratory for microbiome analysis (Wright Labs, Huntingdon, PA, USA).

### 2.7 DNA extraction, library generation, and sequencing

DNA was extracted from samples using the ZymoBIOMICS DNA/RNA Miniprep Kit (Zymo Research, Irvine, CA, USA) according to the manufacturer's protocol and eluted using 50 uL of DNase/RNase free water. After extraction samples were quantified using an Invitrogen Qubit 4 Fluorometer and 1x dsDNA High Sensitivity Assay Kit (ThermoFisher Scientific, Waltham, MA, USA).

Metagenomic libraries were prepared using DNA extracts and the Nextera XT DNA Library Preparation kit (Illumina, San Diego, CA, USA). Libraries were quality checked using an Agilent 2100 Bioanalyzer and DNA High Sensitivity kit and then pooled in an equimolar ratio. The pool was gel purified using a 2% agarose gel and the Qiagen QIAquick gel extraction kit (Qiagen, Germantown, MD, USA). Following purification, the pool was sequenced using an Element AVITI to produce 2 × 150 bp reads.

### 2.8 Bioinformatic preprocessing

Quality was checked for raw data with fastqc (24). sBased on initial quality, fastp (25) was used to filter the data with a sliding window of 4 and an average Phred *q* score of 28 was used. Sequences shorter than 90 bp were discarded.

The remaining sequences were then annotated using Kraken2 (26) with a version of its standard database that included fungi in addition to the standard libraries. A table of species level annotations was then created for use with downstream analyses, with *Homo sapiens* being excluded to avoid human contamination impacting results.

Prior to functional annotation, sequences identified as human were removed, and the remaining

sequences were dereplicated with VSEARCH (27). Emapper v2.0 (28) using the eggNOG 5.0 database (29) was run on the dereplicated sequences. Hits against Kyoto Encyclopedia of Genes and Genomes (KEGG) orthologs were then used, along with the abundances of the sequence they aligned to, to create a table of gene abundances.

### 2.9 Metabolomic analyses

Samples were sent to a third-party laboratory for metabolomic processing and analysis (Creative Proteomics, Shirley, NY, USA). An aliquot of each fecal sample (100 μl) from the metabolite stabilization buffer was lyophilized and extracted with 300 μl cold methanol, followed by vortexing and sonication for 30 min at 4 °C. Samples were stored at −20 °C for 1 h, re-vortexed, and incubated at 4 °C for 30 min. After centrifugation (12,000 rpm, 15 min, 4 °C), the supernatant was collected, stored at −20 °C for 1 h, and centrifuged again under identical conditions. A final 200 μl of supernatant with 5 μl of internal standard (DL-o-chlorophenylalanine, 0.2 mg/ml) was transferred to vials for analysis.

### 2.10 UPLC-MS analysis

Untargeted metabolomics was performed using an ACQUITY UPLC system (Waters) equipped with a HSS T3 column (100 × 2.1 mm, 1.8 μm) and coupled to a Q Exactive Orbitrap mass spectrometer (Thermo Scientific). The mobile phase consisted of 0.05% formic acid in water (solvent A) and acetonitrile (solvent B), delivered via gradient elution. The flow rate was 0.3 ml/min, with the column maintained at 40 °C. Data were acquired in both positive and negative electrospray ionization (ESI) modes using full-scan (m/z 70–1050) and data-dependent MS2 (TopN = 10) modes. Optimized ESI parameters included a spray voltage of 3.0 kV (positive mode) or 3.2 kV (negative mode), with capillary temperature set at 350 °C.

### 2.11 Digestion-associated quality of life questionnaire

The DQLQ was assessed before and after each 14-day supplement period. The DQLQ is a 9-item questionnaire that assesses how often digestive symptoms interfere with quality of life in healthy adults over the past seven days. Each statement is scored as follows: never = 0, rarely = 0.1, occasionally = 0.3; sometimes = 0.5, frequently = 0.7, usually = 0.9, and always = 1.0. The total score represents the sum of the responses to the 9 statements with possible scores range from 0 to 9; a higher score indicates worse digestion associated with quality of life. The DQLQ has been previously shown to be valid and reliable (30).

### 2.12 Statistical analysis

Nutrient intake and nutrient gaps statistical analyses were conducted using GraphPad Prism. Results are expressed as mean ± standard deviation unless otherwise specified. Baseline nutrient intake and EAR values were compared between groups to confirm no significant initial differences. Data distribution was assessed for normality through visual inspection of homoscedasticity and Q–Q plots. A two-way repeated measures ANOVA was applied to examine differences between conditions (AG1^®^ vs. PL) in dietary intake and the total number of EARs achieved. Additionally, two-way ANOVA was performed to evaluate condition-specific differences for individual nutrient EARs. Where significant between-condition effects were observed, pairwise comparisons were carried out to assess within-condition changes from baseline using Fisher's LSD without correction. Sex-specific effects were explored by modeling change scores (endpoint minus baseline) using multiple linear regression with treatment, sex, and a treatment × sex interaction term.

Alpha diversity was calculated by subsampling the table at 10 different depths, ranging from 86,000 to 860,000 sequences for the taxa dataset and 112,000 to 1,120,000 for the KEGG gene dataset. Twenty iterations were performed at each depth to obtain average alpha diversity values for the different metrics [Observed Features and Pielou's Evenness (31)]. Averages for the greatest depth were used to see if any of the alpha diversity metrics differed significantly based on metadata parameters of interest (Wilcoxon Signed-rank Test for comparisons containing two groups and Linear Mixed Effect Models for the comparison among all groups, *p* ≤ 0.05).

Beta diversity analyses were conducted after the tables had first undergone counts per million normalization to mitigate differences between samples based on sequencing depth. Distances between samples were calculated using the Bray-Curtis distance metric (32). The resulting distance matrix was visualized as a Principal Coordinates Analysis plot. Statistical differences between sample groupings were evaluated with individual being used as strata to prevent samples from the same individual being grouped together during randomizations (PERMANOVA, *p* ≤ 0.05). Multilevel Partial Least Squares Discriminant Analysis (PLS-DA) was performed using the mixOmics package (33) through R using Individual (R Core Team 2018) to see if samples clustered distinctly according to categorical metadata groupings of interest.

Biomarker analysis was performed using Linear Discriminant Analysis Effect Size (LEfSe) (34) to identify features that had significantly different abundances based on metadata parameters of interest. For both datasets, the table was normalized with the counts per million method. For taxa and stratified functional features (genes or pathways), only features identified as having significantly differential abundance (Kruskal–Wallis, *p* ≤ 0.05) with a log (LDA) score of at least 1.0 for taxa and 0.25 for functional genes were considered to be enriched. Taxa additionally had to have an average CPM value of at least 10 in the group they were associated with to be considered differentially abundant. Bonferroni corrections were applied to account for multiple statistical testing.

Raw spectral data were processed using Compound Discoverer 3.0 (Thermo Fisher Scientific) for peak alignment and feature detection. Metabolites from both ion modes were merged and imported into SIMCA-P (v14.1, Umetrics) for multivariate analysis. Principal Component Analysis (PCA) was used for data visualization and outlier detection. Supervised analyses, including Partial Least Squares-Discriminant Analysis (PLS-DA) and Orthogonal PLS-DA (OPLS-DA), were performed to identify discriminative metabolites. Biomarkers were selected based on variable importance in projection (VIP > 1.0) and statistical significance (*p* < 0.05, unpaired *t*-test). The robustness of the models was assessed using *R*^2^ (model fit) and *Q*^2^ (predictive ability).

Quality control (QC) samples were generated by pooling equal volumes of each fecal extract and processed identically to individual samples. QC samples were run periodically throughout the analytical sequence to monitor system stability. Over 70% of detected features in QC samples showed a relative standard deviation (RSD) below 30%, confirming acceptable analytical reproducibility. Simple *T*-tests were employed per metabolite, and Bonferroni corrections were applied to account for multiple statistical testing.

DQLQ questionnaire data were analyzed using a two-way repeated measures ANOVA to examine the differences between conditions (AG1^®^ vs. PL) to assess time effects and group by time interactions. Where significant between-condition effects were observed, pairwise comparisons were carried out to assess within-condition changes from baseline using Fisher's LSD without correction. Data were assessed for normality using the Shapiro–Wilk test prior to analyses and all data were normally distributed. Additionally, no adverse events were reported in either AG1^®^ or PL groups during their respective supplementation periods.

## 3 Results

### 3.1 Nutrient gaps and intake results

**Figure 2:**
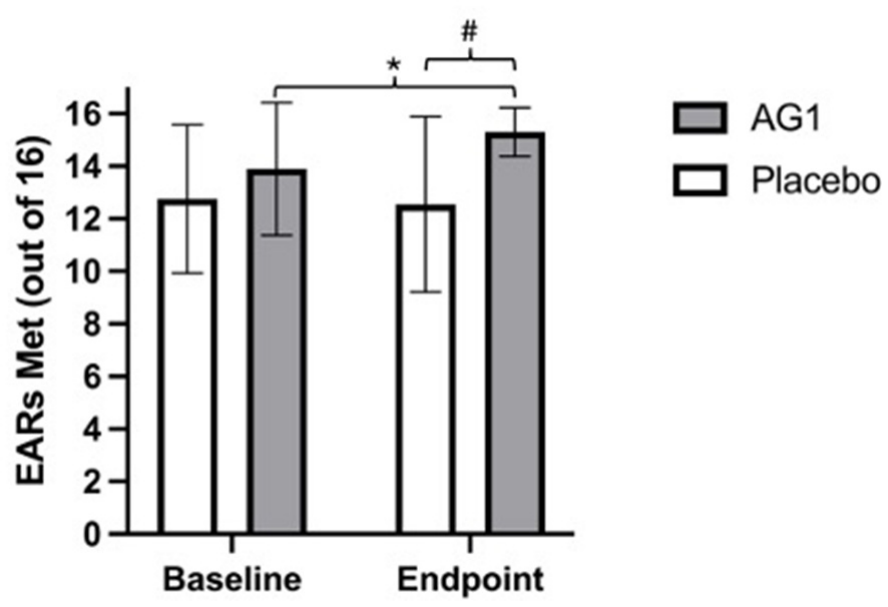
Nutrient gap scores. Data are presented as means ± standard deviations. Scores represent the average number of Estimated Average Requirements (EARs) met, with possible scores ranging from 0 to 16. ^#^significant (*p* = 0.0011) Time × Treatment interaction and endpoint mean difference; *significant (*p* = 0.0159) within condition difference.

All 20 participants completed baseline and endpoint recalls and were included in the nutrient gap analysis. Baseline dietary intake was similar between conditions with no significant differences (*p* > 0.05) observed for any of the reported nutrients. At baseline, the AG1^®^ and placebo groups met 13.9 ± 2.5 and 12.8 ± 2.8 out of 16 nutrient EARs, respectively with no significant difference between groups (*p* > 0.05). Following the intervention, AG1^®^ significantly increased the total number of EARs met compared to placebo [mean difference = 2.8 (95% CI: 1.1, 4.4); *p* = 0.0011] (Figure 2). Within the AG1^®^ group, 1.4 (95% CI: 0.3, 2.5; *p* = 0.0159) nutrient gaps were closed on average compared to −0.2 (95% CI: −1.3, 0.9) in the placebo group. There was no evidence that sex modified the effect of AG1^®^ on nutrient adequacy, as the treatment × sex interaction for change in total EARs met was not significant (*p* = 0.71). Vitamins A, C, and E were the most common nutrient gaps filled by AG1^®^ supplementation (Table 1).

**Table 1:** Nutrient intake changes following baseline to endpoint.

**Self-reported intake, units**	**Intervention**	**Baseline (Day 1)**	**Endpoint (Day 14)**	**Time**	**Group**	**Time × Treatment**
Calories, kcal	AG1	2,433 ± 964	2,272 ± 999	0.3581	0.4748	0.4865
	Placebo	2,179 ± 656	2,157 ± 812			
Protein, g	AG1	133 ± 53	130 ± 71	0.3696	0.559	0.6801
	Placebo	126 ± 55	117 ± 53			
Fat, g	AG1	107 ± 61	93 ± 49	0.2677	0.4398	0.3823
	Placebo	90 ± 31	89 ± 44			
Carbohydrates, g	AG1	236 ± 95	232 ± 105	0.9308	0.4933	0.6514
	Placebo	213 ± 82	219 ± 81			
Sugar, g	AG1	83 ± 37	92 ± 66	0.6945	0.4454	0.5463
	Placebo	77 ± 56	76 ± 43			
Fiber, g	AG1	20 ± 10	22.4 ± 12	0.5482	0.683	0.369
	Placebo	20.1 ± 11.9	19.6 ± 10.7			
Calcium, mg	AG1	1,117 ± 563	1,125 ± 667	0.869	0.5137	0.793
	Placebo	1,033 ± 556	997 ± 496			
Iron, mg	AG1	17.2 ± 6.9	15.5 ± 5.9	0.4652	0.4571	0.2504
	Placebo	14.8 ± 5.6	15.2 ± 5.9			
Magnesium, mg	AG1	376 ± 126	389 ± 183	0.5142	0.7825	0.8897
	Placebo	359 ± 202	379 ± 157			
Phosphorus, mg	AG1	1,887 ± 729	1,872 ± 964	0.4307	0.4613	0.5362
	Placebo	1,777 ± 738	1,646 ± 617			
Potassium, mg	AG1	3,380 ± 1,378	3,641 ± 1,976	0.9159	0.3552	0.224
	Placebo	3,172 ± 1,551	2,952 ± 1,575			
Sodium, mg	AG1	4,265 ± 1,734	3,681 ± 1,616	0.1923	0.621	0.1934
	Placebo	3,763 ± 1,231	3,762 ± 1,395			
Zinc, mg^#^	AG1	15.5 ± 7.6	33.2 ± 6.4^*^	< 0.0001	< 0.0001	< 0.0001
	Placebo	14.4 ± 6.1	15 ± 6.4			
Copper, mg	AG1	1.6 ± 1.1	1.5 ± 0.6	0.6815	0.2308	0.8762
	Placebo	1.3 ± 0.6	1.3 ± 0.5			
Selenium, mcg	AG1	181.9 ± 70.4	188.6 ± 84.9	0.8492	0.1097	0.4352
	Placebo	159.6 ± 54.0	148.6 ± 66.7			
Vitamin C, mg^#^	AG1	122.6 ± 93.8	638.3 ± 128.5^*^	< 0.0001	< 0.0001	< 0.0001
	Placebo	100.5 ± 98.0	103.5 ± 100.7			
Thiamin, mg^#^	AG1	1.7 ± 0.8	4.7 ± 0.8^*^	< 0.0001	< 0.0001	< 0.0001
	Placebo	1.8 ± 0.8	1.6 ± 0.9			
Riboflavin, mg^#^	AG1	2.5 ± 1.0	4.3 ± 1.2^*^	< 0.0001	0.0026	< 0.0001
	Placebo	2.6 ± 1.2	2.5 ± 0.9			
Niacin, mg^#^	AG1	36.4 ± 18.3	53.5 ± 20.8^*^	0.0065	0.0884	0.0006
	Placebo	36.2 ± 19.3	34.0 ± 19.6			
Vitamin B6, mg^#^	AG1	2.8 ± 1.5	8.1 ± 1.8^*^	< 0.0001	< 0.0001	< 0.0001
	Placebo	2.8 ± 1.8	2.7 ± 1.8			
Folate, mcg DFE^#^	AG1	541.4 ± 212.9	1,208.5 ± 223.1^*^	< 0.0001	< 0.0001	< 0.0001
	Placebo	523.7 ± 229.5	553.3 ± 307.5			
Vitamin B12, mcg^#^	AG1	7.2 ± 4.0	406.7 ± 5.0^*^	< 0.0001	< 0.0001	< 0.0001
	Placebo	7.0 ± 5.0	7.2 ± 4.0			
Vitamin A, mcg RAE	AG1	771.6 ± 491.7	1,473.2 ± 625.9^*^	0.0001	0.2726	0.4462
	Placebo	662.5 ± 308.8	1,152.1 ± 1,233.9^*^			
Vitamin E, mg alpa-tocopherol^#^	AG1	11.9 ± 5.6	32.2 ± 8.5^*^	< 0.0001	0.0001	< 0.0001
	Placebo	11.1 ± 6.3	13.1 ± 12.9			
Choline, mg	AG1	637.9 ± 302.5	591.0 ± 338.3	0.4891	0.3825	0.7019
	Placebo	547.5 ± 261.5	533.9 ± 282.0			
Vitamin D, mcg D2 + D3	AG1	10.1 ± 10.4	9.8 ± 14.7	0.9393	0.7137	0.8083
	Placebo	8.3 ± 11.8	8.9 ± 13.4			
Lutein + Zeaxanthin, mcg	AG1	2,232 ± 2176	1,831 ± 1611	0.2553	0.8094	0.0298
	Placebo	1,553 ± 1321	2,793 ± 3113			
Vitamin K, mcg phylloquinone	AG1	158.4 ± 162.4	130.1 ± 109.5	0.2968	0.9533	0.0522
	Placebo	96.4 ± 65.1	187.9 ± 205.4^*^			
Cholesterol, mg	AG1	749 ± 440	621 ± 452	0.3215	0.3487	0.3857
	Placebo	590 ± 305	582 ± 356			
Saturated fat, g	AG1	36.0 ± 26.7	28.8 ± 17.8	0.4001	0.5359	0.2096
	Placebo	28.5 ± 10.5	29.9 ± 19.5			
Monounsaturated fat, g	AG1	35.6 ± 20.8	31.7 ± 17.1	0.2809	0.6288	0.6301
	Placebo	32.1 ± 12.4	30.7 ± 14.1			
Polyunsaturated fat, g	AG1	23.6 ± 13.0	20.1 ± 10.3	0.1196	0.2285	0.6888
	Placebo	19.3 ± 8.8	17.2 ± 11.3			

Mean ± standard deviation dietary intake of selected nutrients assessed using ASA24^®^ (*n* = 20). Values are expressed per day. Nutrient values from AG1^®^ or placebo were added to the respective endpoint diets except vitamin K (ASA24^®^ reports phylloquinone; AG1^®^ provides MK-7), so AG1^®^ vitamin K was not added. Bolded nutrients are those provided in the study product, whereas italicized nutrients are not contained in the study product. ^*^Significant (*p* < 0.05) within condition differences; #Significant (*p* < 0.05) endpoint mean differences.

### 3.2 Digestion-associated quality of life questionnaire

All 20 participants completed baseline and endpoint questionnaires and were included in the DQLQ analysis. Baseline DQLQ scores were similar between conditions with no significant differences (*p* = 0.919) observed. There was no significant main effect for time (*p* = 0.272) over the 2-week intervention. Additionally, there was no group by time interaction between AG1^®^ and PL over the course of the 2-week intervention (*p* = 0.777).

### 3.3 Sequencing results

A total of 79 stool samples were collected and processed. All samples yielded high-quality sequence depth with >1.5 million sequences per sample. The mean sequencing depth was 8.24 million sequences, with the range being between 4.4 million and 12.6 million sequences observed. A histogram representing the distribution of sequence counts can be found in Supplementary Figure 2.

### 3.4 Community structure

**Figure 3:**
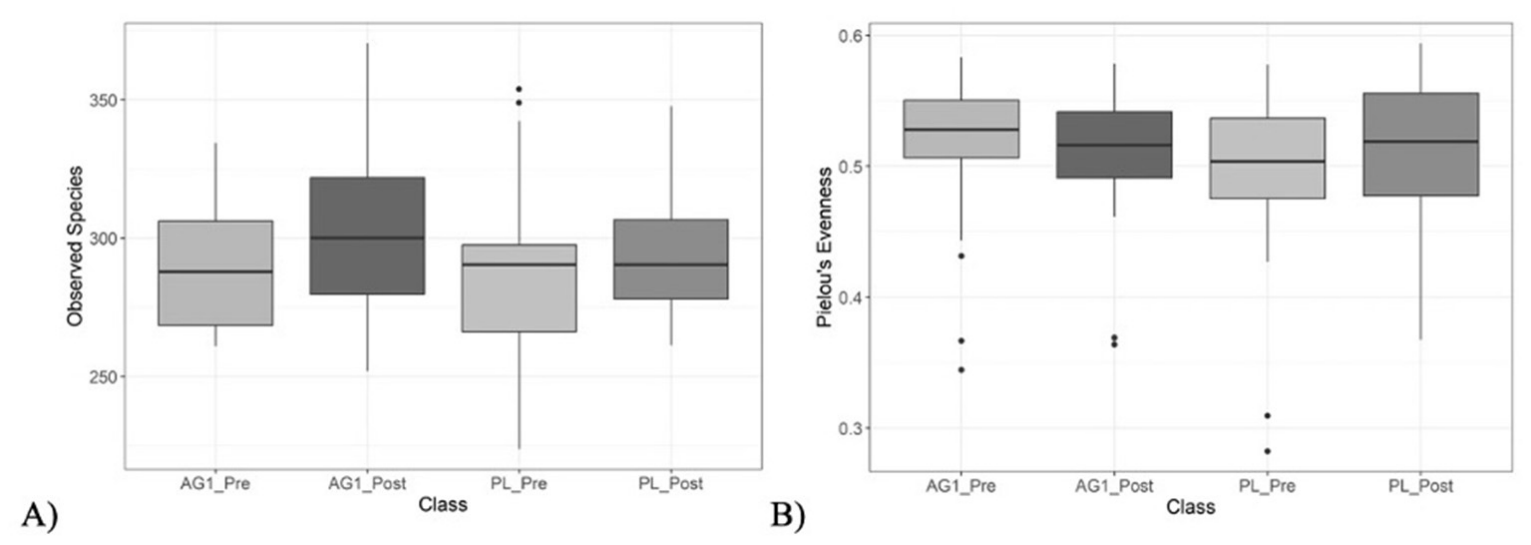
Species richness and evenness were measured and visualized using box and whisker plots. **(A)** Species richness was measured using the total number of observed species, with no significant pairwise differences observed (*p* > 0.05). **(B)** Species evenness was measured using Pielou's evenness alpha diversity metric, with no significant pairwise differences observed (*p* > 0.05).

Changes in alpha diversity were determined using the total number of observed species (species richness, Figure 3A) as well as Pielou's evenness (species evenness, Figure 3B). The mean number of species observed was not significantly (*p* > 0.05) altered in any cohort regardless of treatment or timepoint. Slight variations in richness and evenness metrics were observed across groups, but the differences were not statistically significant.

**Figure 4:**
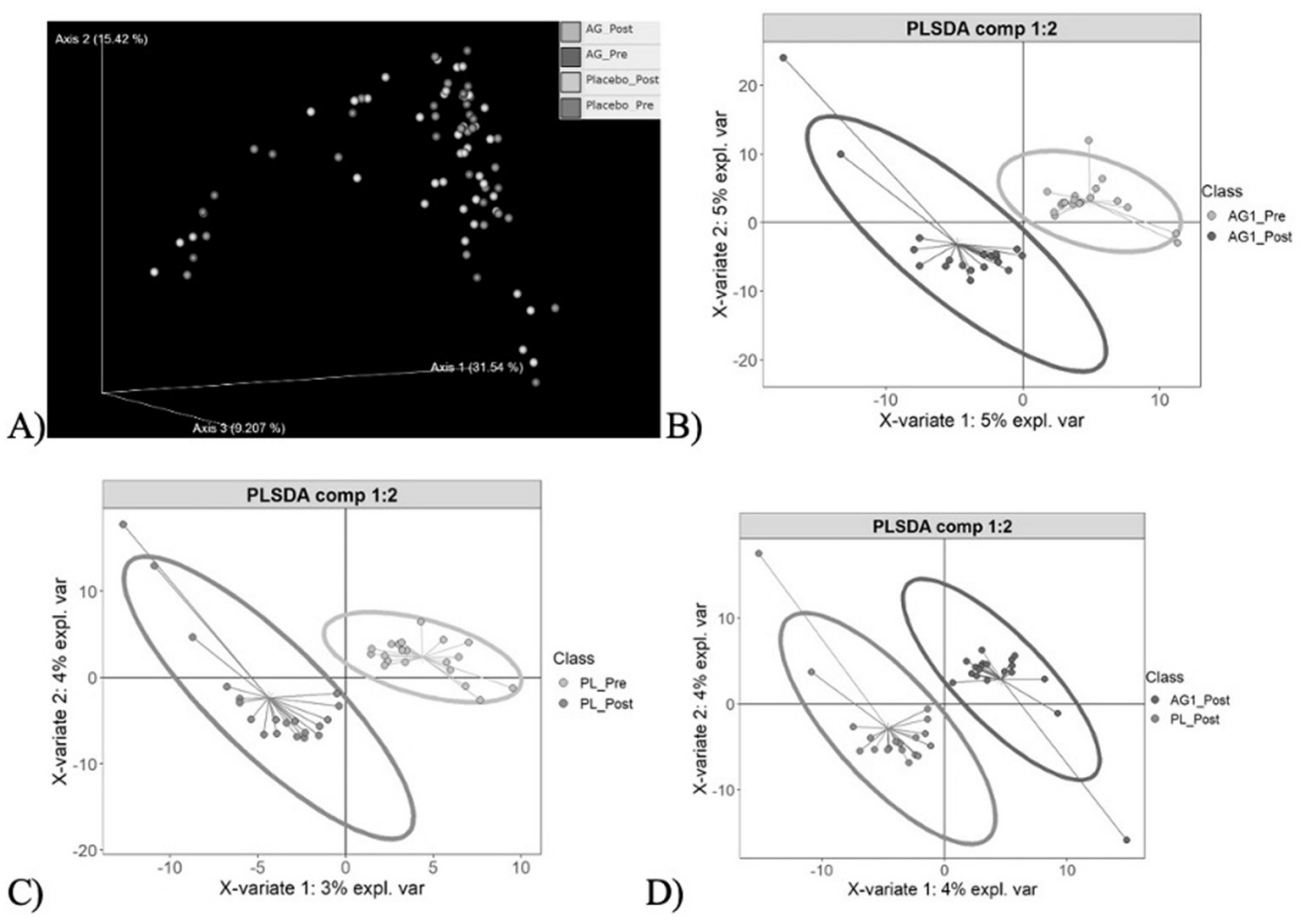
Community heterogeneity visualization. **(A)** Principle Coordinate Analysis (PCoA) plot displays clustering of all four sample cohorts based on the Bray-Curtis dissimilarity metric, with no significant clustering observed (PERMANOVA, *p* > 0.05). **(B–D)** Pairwise Partial Least Squares Discriminant Analysis (PLS-DA) plots highlight differences in microbial composition resulting from AG1, placebo, and at the end point for both treatments respectively.

Community heterogeneity was visualized using Principle Coordinate Analysis (PCoA) and Partial Least Squares Discriminant Analysis (PLS-DA) (Figure 4). The PCoA (Figure 4A) demonstrated clustering amongst the four sample cohorts, with no significant difference in community composition observed (PERMANOVA, *p* > 0.05). These results suggested that both treatment and placebo had no global phylogenetic shift effect on community composition. Further pairwise visualizations were performed (Figures 4B–D). Clear clustering between the pre and post treatment of AG1^®^ was observed alongside a greater variance resulting (Figure 4B) relative to the placebo treatment (Figure 4C) suggesting AG1^®^ had greater impacts on community composition despite there not being statistically significant. Such findings warranted follow-up biomarker discovery to determine what taxa were significantly impacted by AG1^®^ treatment.

**Figure 5:**
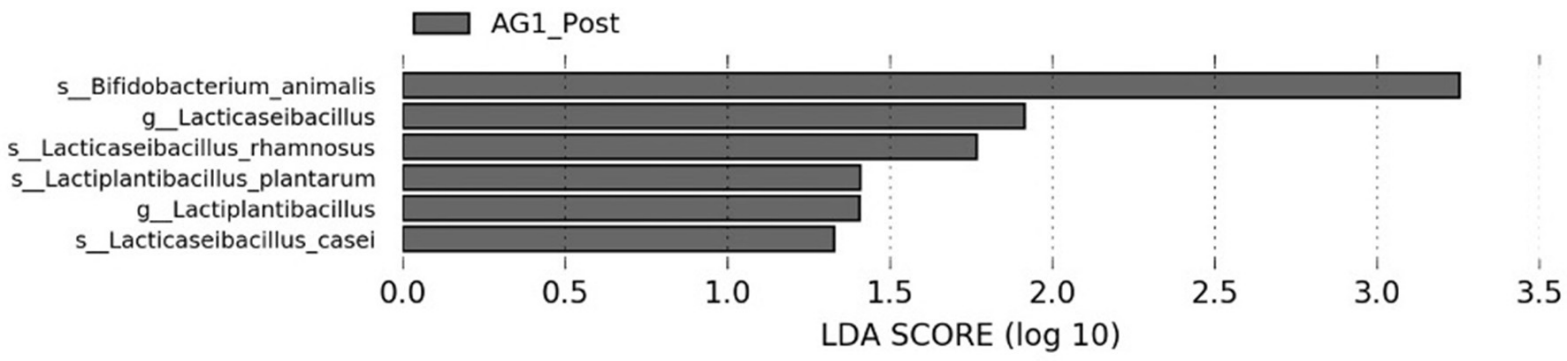
LEfSe bar plot demonstrates significantly enriched biomarker taxa (LDA > 1.0, p < 0.05) that were identified when comparing both treatment groups at baseline and endpoint. Six biomarker taxa were identified, and all belong to the post AG1^®^ treatment group. No biomarker taxa were observed for the placebo group or at baseline prior to AG1^®^ supplementation.

Biomarker discovery was performed using LEfSe for the AG1^®^ treatment. When exploring the changes in specific bacterial taxa between the two treatment groups, six taxa were differentially enriched after AG1^®^ treatment (Figure 5). Of the taxa, two were at the genus level (*Lacticaseibacillus* and *Lactiplantibacillus*). The four species that were enriched included *Lacticaseibacillus casei, Lactiplantibacillus plantarum, Lacticaseibacillus rhamnosus*, and *Bifidobacterium animalis*. All observed enriched taxa were biologically meaningful (LDA score > 1) and statistically significant (*p* < 0.05).

**Figure 6:**
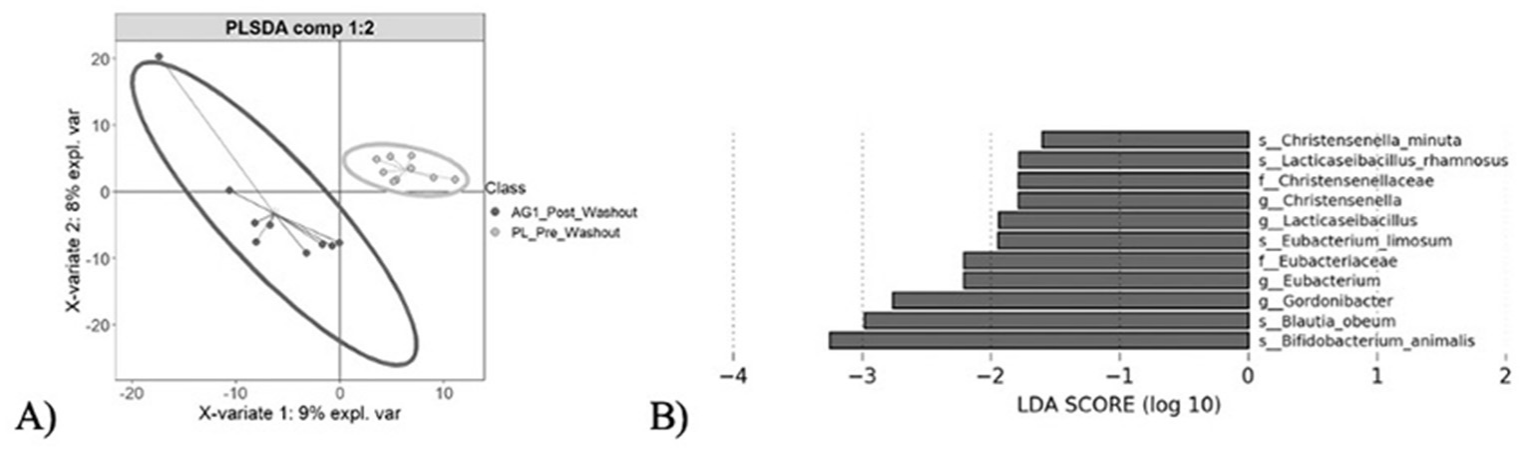
Partial Least Squared Discriminant Analysis (PLS-DA) ordination and subsequent taxa biomarker discovery demonstrated significant reductions in gut microbial changes following AG1 treatment cessation. **(A)** Post AG1^®^ treatment samples distinctly clustered away from the same microbial communities following a two-week washout period. **(B)** Taxa biomarker discovery demonstrated a total of eleven taxonomic groups that significantly diminished (LDA score < −1, *p* < 0.05) following AG1^®^ treatment cessation. No groups were enriched following treatment suggesting gut community changes diminished as opposed to shifted following treatment cessation.

A *post-hoc* exploration was performed to look at individuals who took AG1^®^ prior to placebo which elucidated what happens after the two-week washout period following AG1^®^ cessation (Figure 6). PLS-DA plotting demonstrated clear clustering (Figure 6A) which warranted subsequent taxa biomarker discovery. A total of 11 taxa was differentially enriched after AG1^®^ treatment relative to the subsequent baseline measurement (Figure 6B). These taxa included two families (Christensenellaceae and Eubacteriaceae), four genera (*Christensenella, Lacticaseibacillus, Eubacterium*, and *Gordonibacter*), and five species (*Christensenella minuta, Lacticaseibacillus rhamnosus, Eubacterium limosum, Blautia obeum*, and *Bifidobacterium animalis*).

### 3.5 Putative community function

**Figure 7:**
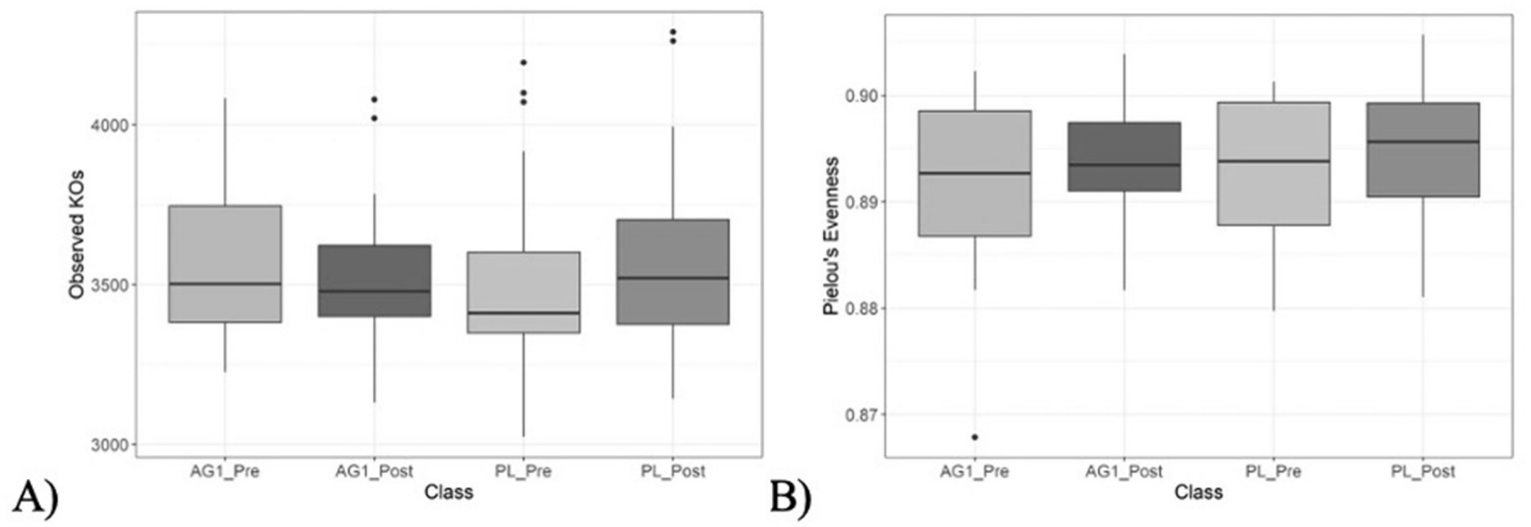
KEGG gene richness and evenness were measured and visualized using box and whisker plots. **(A)** KEGG gene richness was measured using the total number of observed species, with no significant pairwise differences observed (*p* > 0.05). **(B)** KEGG gene evenness was measured using Pielou's evenness alpha diversity metric, with no significant pairwise differences observed (*p* > 0.05).

Changes in putative community metagenomic functionality data was observed. Changes in the total number of observed KEGG genes (gene richness, Figure 7A) and KEGG gene evenness (Pielou's Evenness, Figure 7B) did not yield significant alterations following AG1^®^ treatment. The mean number of KEGG genes observed nor the evenness in their representation was not significantly (*p* > 0.05) altered in any cohort regardless of treatment or timepoint. Based on the similar analysis but at the taxonomic level, these findings were not surprising.

**Figure 8:**
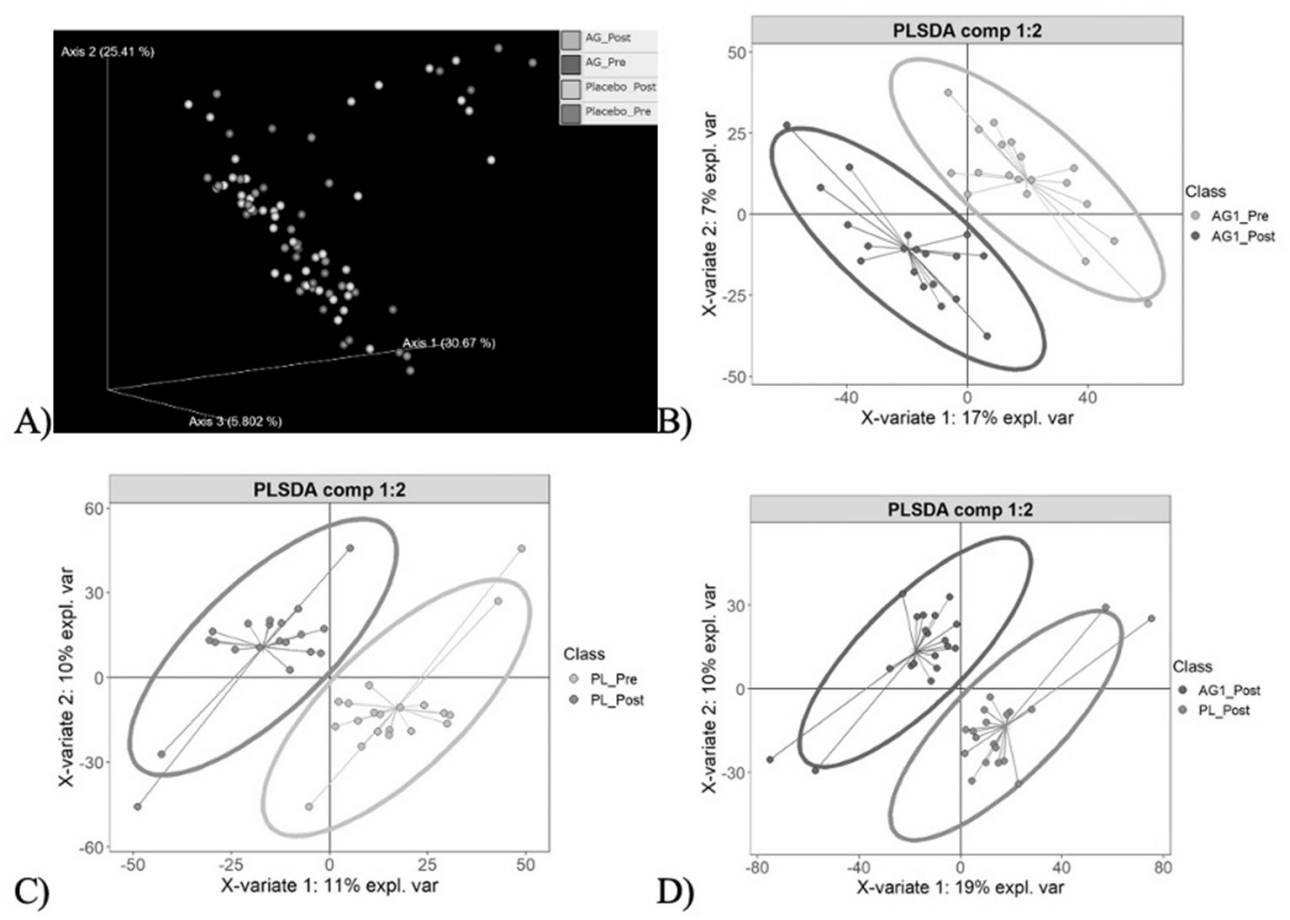
Community genetic heterogeneity visualized using PCoA and PLS-DA. **(A)** PCoA demonstrating clustering amongst the four sample cohorts, with no significant difference in KEGG gene composition observed (PERMANOVA, *P* > 0.05). These results suggested that both treatment and placebo had no global shift effect on the community's number of genes. **(B–D)**. Further pairwise visualizations showing clear clustering between the pre and post treatment of AG1^®^ was observed alongside a greater variance resulting relative to the placebo treatment suggesting AG1^®^ had greater impacts on the relative KEGG gene counts within the community despite not being statistically significant.

Community genetic heterogeneity was visualized using PCoA and PLS-DA (Figure 8). The PCoA (Figure 8A) demonstrated clustering amongst the four sample cohorts, with no significant difference in KEGG gene composition observed (PERMANOVA, *p* > 0.05). These results suggested that both treatment and placebo had no global shift effect on the community's number of genes. Further pairwise visualizations were performed (Figures 8B–D). Clear clustering between the pre and post treatment of AG1^®^ was observed alongside a greater variance resulting (Figure 8B) relative to the placebo treatment (Figure 8C) suggesting AG1^®^ had greater impacts on the relative KEGG gene counts within the community despite not being statistically significant. Such findings warranted follow-up biomarker discovery to determine what genes were significantly impacted by AG1^®^ treatment.

Based on the significant shifts in the six aforementioned taxa, functional pathway analysis was conducted to elucidate the biological impact the shift in these taxa might have on the host. The identified KEGG genes were functionally annotated and the top 20 functional pathways were explored (Supplementary Figure 3). Globally, these pathways contribute to normal microbial metabolic function but can also make contributions to the host's gut health and influence microbial nutrient transport and metabolism.

Broader gene biomarker discovery was conducted using LEfSe for the AG1 treatment. A total of 294 KEGG genes were significantly enriched among the cohort, with all 294 genes exclusively detected in the post AG1^®^ treatment group. Notably, 288 of these orthologs were mapped to the *B. animalis* genome but likely stems from the magnitude of gut enrichment of this microbe from the AG1^®^ treatment. The top 10 KEGG pathways associated with these genes accounted for 107 of the mapped enriched gene counts (Supplementary Table 1).

Subsequent metabolite predictions were conducted from two treatments. Unfortunately, no metabolites were predicted to be enriched amongst any of the treatment groups despite the changes in biomarker KEGG genes. This is likely due to the depth of sequencing causing genomic content associated with not expressed genes to induce too much genetic noise to draw meaningful conclusions. Therefore, next step experiments should include transcriptomics and/or metabolomics to understand the consequences of these changes in KEGG orthologs.

KEGG gene biomarker discovery was also conducted on individuals who took AG1^®^ prior to placebo. PLS-DA ordination shows distinct clustering between the post AG1^®^ treatment group and the period following a two-week washout (Supplementary Figure 4). A total of 8 KEGG orthologs were discovered to be diminished following AG1^®^ treatment cessation. Four of the orthologs are associated with *B. animalis*, supporting the notion that treatment cessation causes a reduction in the potential gut health benefits AG1^®^ yields (Supplementary Table 2).

### 3.6 Metabolomics results

Full metabolomic results are presented in Supplementary Tables 3, 4. Briefly, AG1^®^ supplementation led to significant changes in fecal metabolite profiles, as determined by untargeted UPLC-MS analysis. Among increased metabolites, mevalonic acid (*p* = 0.01) was elevated, corresponding to activity in the terpenoid backbone biosynthesis pathway. Genistein (*p* = 0.04), a polyphenolic isoflavone, and medicagenic acid (*p* = 0.01), a plant-derived triterpenoid, also increased. Additional increases were observed in 9,10-dihydroxyoctadecanoate (*p* = 0.02), a hydroxy-fatty acid, and pyrimidine (*p* = 0.01), a nucleotide base precursor. Several bile acid and cholesterol intermediates were significantly elevated, including 3α,7α-dihydroxy-5β-cholestane (*p* = 0.03), 4α-carboxy-5α-cholesta-8-en-3β-ol (*p* = 0.04), and 27-nor-5β-cholestane-3α,7α,12α,24,25-pentol (*p* = 0.01).

Significant reductions were detected in compounds associated with protein fermentation and environmental exposure. These included piperidine (*p* = 0.03), 4-aminophenol (*p* = 0.03), and N,N-dimethyl-p-phenylenediamine (*p* = 0.01). The caffeine metabolite 3-methylxanthine was also decreased (*p* = 0.03). Additional reductions occurred in xanthurenic acid (*p* = 0.048), associated with tryptophan metabolism, and deoxyribose (*p* = 0.02), a pentose phosphate pathway product. The secondary bile acid 12-ketodeoxycholic acid was significantly decreased (*p* = 0.03), suggesting altered microbial bile acid metabolism.

## 4 Discussion

The present study examined the effects of 2 weeks of AG1^®^ supplementation on gut microbial composition, nutritional adequacy, and tolerability in resistance-trained adults using a randomized, double-blind, placebo-controlled crossover design. The primary findings indicate that AG1^®^ supplementation significantly improved nutritional adequacy by increasing the number of micronutrient EARs met, without negatively affecting digestive quality of life. While AG1^®^ did not produce large, global shifts in microbial alpha or beta diversity, supplementation was associated with selective enrichment of several bacterial taxa commonly linked to gut health, including *Lactiplantibacillus plantarum, Lacticaseibacillus rhamnosus*, and *Bifidobacterium animalis*. These findings suggest that short-term intake of the multi-ingredient nutritional supplement can meaningfully improve dietary micronutrient sufficiency while improving gut microbial composition, without disrupting overall community structure in a healthy, physically active population.

A central finding of this investigation was that AG1^®^ supplementation significantly improved dietary micronutrient adequacy, as evidenced by an increase in the total number of EARs met compared with placebo. Despite being resistance-trained and a generally health-conscious, participants exhibited baseline gaps in several micronutrients, reinforcing prior observations that active individuals may still fail to meet recommended intakes through habitual diet alone (35). Tinsley et al. (35) recently identified the presence of several nutrient gaps among exercising adults, with common inadequacies including calcium, folate, magnesium, and vitamins A, C, D, and E among females, and vitamins C, D, and E among males. In the current study, the most frequently closed gaps following AG1^®^ supplementation were vitamins A, C, and E, nutrients that play critical roles in immune function, antioxidant defense, and cellular health (36–38). These findings support the concept that multi-ingredient nutritional supplements can serve as an effective adjunct to the diet by helping close micronutrient gaps when whole-food intake alone is insufficient to consistently meet established dietary targets. Importantly, improvements in nutrient adequacy occurred without meaningful changes in total energy intake or digestive quality of life. Collectively, these results highlighting the importance of assessing dietary adequacy using EAR-based approaches and demonstrate the potential utility of nutrition supplements for enhancing micronutrient sufficiency in physically active populations.

In addition to improving dietary micronutrient adequacy, AG1^®^ supplementation elicited targeted, meaningful changes in gut microbiome composition without inducing broad disruptions to community structure. Consistent with other studies in a healthy cohort, no significant alterations were observed in global alpha or beta diversity metrics following supplementation (39, 40). However, biomarker discovery analyses revealed enrichment of several probiotic-associated taxa, including *L. casei, L. plantarum, L. rhamnosus*, and *B. animalis*, species that have been previously linked to gastrointestinal health, immune modulation, and metabolic function (41–43). At the functional level, AG1^®^ supplementation was associated with enrichment of KEGG orthologs and pathways involved in core microbial metabolic processes, largely driven by increased abundance of *B. animalis*. Although global functional diversity metrics were unchanged, the exclusive detection of enriched functional genes following AG1^®^ supplementation indicates a shift in the metabolic potential of the gut microbiome toward pathways relevant to nutrient transport and microbial metabolism. Notably, cessation of supplementation was accompanied by reductions in both specific taxa and associated functional genes, supporting the notion that continued intake may be necessary to sustain these microbiome-related effects. Collectively, these results align with prior *in vitro* and human studies demonstrating that AG1^®^ can induce subtle yet functionally relevant microbiome adaptations without compromising community stability or gastrointestinal tolerability (14–17, 19).

Exploratory metabolomic analyses indicated AG1^®^ supplementation led to changes in stool metabolites associated with altered host and microbiome function following the 14-day intervention. Increases in genistein and mevalonic acid may suggest enhanced antioxidant capacity, isoprenoid synthesis, and cardiometabolic support (44–46). Decreases in xenobiotic compounds such as N,N-dimethyl-p-phenylenediamine, and markers of microbial proteolysis (e.g., piperidine, 4-aminophenol) imply reduced toxicant burden and proteolytic fermentation (47–49). Many of the metabolite changes plausibly relate to the shifts in gut microbial composition caused by AG1^®^. The enrichment of probiotic taxa (e.g., *B. animalis, L. rhamnosus, L. plantarum*) provides a mechanistic basis for some of the observed stool metabolites. For example, the rise in 9,10-DHOME may be linked to *Lactobacillus* and *Bifidobacterium* activity. Zhou and colleagues (50) found that *Lactobacillus*-enriched environments (with American ginseng) in a rodent model increased 9,10-DHOME alongside improved gut barrier function, while data stemming from a piglet model suggests it may be indicative of lipid peroxidation (51). It is possible that the *Lacticaseibacillus* and *Bifidobacterium* probiotics in AG1^®^ could be involved in linoleic acid metabolism to DHOME, suggesting a functional change in fatty acid processing by the new microbiome (50, 52). Overall, the metabolomic and metagenomic data together indicate that AG1^®^ supplementation shifted the gut ecosystem toward fiber-fermenting taxa, which in turn led to measurable changes in metabolites. Many of these shifts (e.g., more polyphenols like genistein, fewer harmful protein metabolites) are consistent with improved gut health, whereas others (e.g., increased DHOME) highlight areas requiring further study to determine the role of these metabolites in human models.

An important finding of the present study is that 2 weeks of AG1^®^ supplementation was well tolerated and did not adversely affect digestion-associated quality of life in resistance-trained adults. DQLQ scores remained stable across the intervention period, with no main effects of time or condition and no group-by-time interactions observed between AG1^®^ and placebo. The favorable tolerability profile observed in the present investigation aligns with prior *in vitro* and human studies reporting high digestibility and gastrointestinal safety of AG1^®^ supplementation (18, 19). La Monica et al. (19) similarly observed no statistically significant changes in DQLQ scores following 4 weeks of AG1^®^ supplementation in healthy adults; however a trend (*p* = 0.058) suggested a greater average improvement in DQLQ scores with AG1^®^ compared with a reduction observed in the placebo group. In addition, participants reported no adverse changes in digestive symptoms, bowel movement frequency, or stool consistency, as assessed by subjective questionnaires and the Bristol stool chart (19). Collectively, results from the present investigation and those of La Monica et al. (19) indicate that AG1^®^ supplementation can support nutritional adequacy and enrich beneficial gut microbial taxa without concomitant increases in gastrointestinal symptoms in healthy individuals. The favorable tolerability profile is particularly relevant for physically active individuals, for whom gastrointestinal discomfort may impair training quality, performance, and long-term adherence to supplementation strategies.

This study has several limitations that should be considered when interpreting the findings. First, the relatively small sample size, while comparable to other crossover microbiome interventions, may have limited statistical power to detect subtle changes in microbial diversity. Additionally, the short 14-day intervention period may not have been sufficient to fully capture longer-term adaptations in gut microbiome composition or function, particularly in a healthy, resistance-trained population. Although dietary intake was assessed using multiple 24-h recalls (one at each timepoint) and participants were instructed to maintain habitual dietary patterns, unmeasured day-to-day dietary variability may have influenced microbiome outcomes and may not be an accurate representation of usual dietary intake. Additionally, while metagenomic sequencing provided insight into microbial taxonomic and functional potential, transcriptomic or targeted metabolomic analyses were not performed. Furthermore, although maltodextrin is widely used as a placebo in nutrition research, emerging evidence suggests it may exert modest effects on the gut microbiome (53), and future investigations may benefit from alternative placebo formulations when evaluating microbial composition and function. Finally, the study cohort consisted of young, resistance-trained adults, which limits generalizability to populations such as older adults, sedentary populations, or individuals with gastrointestinal or metabolic disorders.

## 5 Conclusion

In conclusion, 2 weeks of AG1^®^ supplementation meaningfully improved micronutrient adequacy in healthy resistance-trained adults by reducing overall nutrient gaps, with vitamins A, C, and E representing the most frequently addressed deficiencies. In parallel, supplementation selectively enriched key beneficial gut microbial taxa and putative microbial functional without inducing major disruptions to overall community structure. Importantly, AG1^®^ was well tolerated and did not negatively impact digestion-associated quality of life. The findings support the potential role of comprehensive, multi-ingredient nutritional supplements as a practical strategy to enhance nutrition and gut health in physically active individuals. Future research incorporating longer intervention periods, larger sample sizes, and integrated multi-omics approaches will be necessary to further elucidate the durability, functional relevance, and clinical implications of these microbiome adaptations.

## Data Availability

The data presented in this study are publicly available. The data can be found here: https://www.ncbi.nlm.nih.gov, accession PRJNA1441102; https://massive.ucsd.edu, project ID MSV000101264.
