# Supplementary material for: Effect of AG1® supplementation on nutritional adequacy and gut microbial composition in trained adults
Source: Front Nutr. 2026 Mar 31;13:1783951. doi: 10.3389/fnut.2026.1783951 (PMC13077853; doi:10.3389/fnut.2026.1783951)
Supplement: Supplementary file 1 [file Supplementary_file_1.zip › Supplementary Table 3.DOCX]

**Supplementary Table 3.** Significantly altered metabolites which carry positive charges when ionized. Pre and Post values only pertain to AG1^®^ treated individuals to minimize metabolite changes irrelevant to the treatment effect. All units are arbitrary intensity units since untargeted metabolomics was conducted. P values are derived from paired T tests.

| **Metabolite Name** | **Average Pre Value** | **Average Post Value** | **P Value** | **Potential Biochemical Meaning** |
| --- | --- | --- | --- | --- |
| Piperidine | 199.0889836 | 110.9329483 | P = 0.033 | Altered amine metabolism |
| Benzaldehyde | 183.7711289 | 332.5056407 | P = 0.039 | Increased phenylalanine metabolism |
| 4-Aminophenol | 108.6633742 | 60.37271349 | P = 0.028 | Unclear |
| 4-Mercaptopyridine | 17.51711246 | 66.42882148 | P = 0.014 | Unclear |
| Pyrimidine | 161.1256944 | 488.3814437 | P = 0.01 | Increased breakdown of nucleic acids |
| 4-Methyl-5-vinylthiazole | 5.734710875 | 21.48249046 | P = 0.009 | Change in microbiome functionality |
| 5-(1-Hydroxyethyl)-4-methylthiazole | 51.23728248 | 169.2315225 | P = 0.01 | Change in microbiome functionality |
| N,N-Dimethyl-p-phenylenediamine | 80.97954898 | 44.63965436 | P = 0.006 | Change in oxidative stress |
| Nicotine | 17.81986584 | 33.72021094 | P = 0.009 | Unclear |
| 3-Hydroxy-2-methyl-2-vinylcyclohexanone | 71.96984542 | 115.0016261 | P = 0.011 | Altered metabolism of plant material |
| 9-Hydroxymegastigma-4,6,7-trien-3-one | 274.4134371 | 196.8652354 | P = 0.042 | Altered metabolism of carotenoids |
| 3-Oxo-alpha-ionol | 190.7737113 | 136.7304993 | P = 0.023 | Altered metabolism of carotenoids |
| Macaridine | 9.37522694 | 18.06523376 | P = 0.033 | Altered gut microbiome composition |
| 4,8,12-Trimethyl-1,3,7,11-tridecatetraene | 156.2505772 | 216.264731 | P = 0.013 | Likely plant derived |
| 1,8,11,14-Heptadecatetraene | 242.6749497 | 315.9904992 | P = 0.047 | Likely plant derived |
| 2-Hydroxyacorenone | 81.63061113 | 51.94377974 | P = 0.034 | Unclear |
| Phenylalanylproline | 34.67227096 | 13.63611404 | P = 0.015 | Altered peptide metabolism |
| Genistein | 11.75011163 | 20.76433129 | P = 0.045 | Altered metabolism of plant material |
| 12-oxo Phytodienoic Acid | 50.60274157 | 36.53870393 | P = 0.027 | Altered plant metabolism |
| 3a,7a-Dihydroxy-5b-cholestane | 16.17648373 | 27.17301433 | P = 0.027 | Altered bile acid metabolism |
| 4alpha-Carboxy-5alpha-cholesta-8-en-3beta-ol | 40.82458799 | 65.20590894 | P = 0.037 | Altered cholesterol metabolism |
| Arginine-betaxanthin | 274.4353778 | 156.9613734 | P = 0.03 | Altered metabolism of plant material |
| Physapubenolide | 69.53222741 | 139.2569429 | P = 0.019 | Altered metabolism of plant material |
| Ciclesonide | 9.216060374 | 3.559810632 | P = 0.02 | Unclear |
