# Supplementary material for: Effect of AG1® supplementation on nutritional adequacy and gut microbial composition in trained adults
Source: Front Nutr. 2026 Mar 31;13:1783951. doi: 10.3389/fnut.2026.1783951 (PMC13077853; doi:10.3389/fnut.2026.1783951)
Supplement: Supplementary file 1 [file Supplementary_file_1.zip › Supplement Facts.DOCX]

|  | **Supplement Facts** | | | |  |
| --- | --- | --- | --- | --- | --- |
|  | **Serving Size: 1 Level AG1^®^ Scoop^ (~ 1 ½ tbsp) (13g)** | |  |  |  |
|  | **Servings per Container: 30** | |  |  |  |
|  |  |  |  |  |  |
|  |  |  | **Amount Per Serving** | **%DV** |  |
|  |  |  |  |  |  |
|  | Calories | | 40 |  |  |
|  | Total Fat | | 1 g | 1%** |  |
|  | Total Carbohydrate | | 6 g | 2%** |  |
|  |  | Dietary Fiber | 2 g | 7%** |  |
|  |  | Total Sugars | <1 g | † |  |
|  | Protein | | 2 g |  |  |
|  | Vitamin A (as beta-carotene) | | 550 mcg RAE | 61% |  |
|  | Vitamin C (as ascorbic acid) | | 500 mg | 556% |  |
|  | Vitamin E (as mixed tocopherols) | | 20 mg | 133% |  |
|  | Thiamin (Vitamin B1) (as thiamine hydrochloride) | | 3 mg | 250% |  |
|  | Riboflavin (Vitamin B2) | | 2 mg | 154% |  |
|  | Niacin (as nicotinic acid, niacinamide) | | 16 mg NE | 100% |  |
|  | Vitamin B6 (as pyridoxal-5-phosphate (P5P)) | | 5 mg | 294% |  |
|  | Folate (as 5-MTHF) | | 680 mcg DFE | 170% |  |
|  | Vitamin B12 (as methylcobalamin) | | 400 mcg | 16667% |  |
|  | Biotin (as D-biotin) | | 330 mcg | 1100% |  |
|  | Pantothenic acid (Vitamin B5) (as calcium pantothenate) | | 4 mg | 80% |  |
|  | Choline (as choline bitartrate) | | 25 mg | 5% |  |
|  | Calcium (as calcium citrate) | | 120 mg | 9% |  |
|  | Iron | | 1 mg | 6% |  |
|  | Phosphorus | | 130 mg | 10% |  |
|  | Magnesium (as dimagnesium malate, magnesium glycinate) | | 30 mg | 7% |  |
|  | Zinc (as zinc citrate) | | 20 mg | 182% |  |
|  | Selenium (as selenized yeast) | | 22 mcg | 40% |  |
|  | Copper (as copper gluconate) | | 0.2 mg | 22% |  |
|  | Manganese (as amino acid chelate) | | 0.4 mg | 17% |  |
|  | Chromium (as chromium picolinate) | | 25 mcg | 71% |  |
|  | Molybdenum (as molybdenum glycinate) | | 45 mcg | 100% |  |
|  | Sodium | | 40 mg | 2% |  |
|  | Potassium (as dipotassium phosphate) | | 250 mg | 5% |  |
|  |  |  |  |  |  |
|  | Vitamin K2 (as menaquinone-7) | | 90 mcg | † |  |
|  | Benfotiamine | | 25 mg | † |  |
|  | Myo-Inositol | | 100 mg | † |  |
|  | Boron (as boron glycinate) | | 500 mcg | † |  |
|  | Coenzyme Q10 (as ubiquinone) | | 60 mg | † |  |
|  | Alpha-lipoic acid (ALA) | | 100 mg | † |  |
|  | Active Superfood and Prebiotic Complex | | 7.5 g | † |  |
|  |  | Organic apple powder, pea protein isolate, organic spirulina, lecithin (>65% phospholipids), inulin (chicory root) powder, citrus bioflavonoids extract, organic chlorella powder, ginger root powder, cocoa bean powder, licorice root powder, bromelain, beta glucan, artichoke aerial parts (15:1) extract, slippery elm bark powder, rhodiola root (15:1) extract, astragalus root (4:1) extract, cocoa seed extract, organic matcha leaf powder, ashwagandha root (5:1) extract, dandelion whole herb (4:1) extract, eleuthero root (10:1) extract |  |  |  |
|  | Daily Phytonutrient Complex | | 1.5 g | **†** |  |
|  |  | Broccoli powder, papaya fruit powder, beet root powder, organic wheat grass leaf powder, organic alfalfa leaf powder, organic barley leaf powder, carrot root powder, acerola fruit (4:1) extract, rosehip fruit (4:1) extract, pineapple fruit powder, shiitake mushroom powder, reishi mushroom powder, rutin (from *Sophora japonica* bud extract), rosemary leaf (4:1) extract, bladderwrack whole plant powder, wolfberry (*Lycium barbarum*) fruit (4:1) extract, burdock root (4:1) extract, hawthorn berry fruit (10:1) extract, bilberry fruit (100:1) extract, milk thistle seed (70:1) extract, grape seed (120:1) extract |  |  |  |
|  | Dairy-Free Probiotic Blend | | 37 mg (10 Billion Viable Cells^††^) | **†** |  |
|  |  | *Lactobacillus rhamnosus* GG*, Lactobacillus acidophilus* NCFM, *Bifidobacterium lactis* HN019, *Lactobacillus casei* LC-11*, Lactobacillus plantarum* LP-115 |  |  |  |
|  |  |  |  |  |  |
|  | **** Percent Daily Values (DV) are based on a 2,000 calorie diet.** | | |  |  |
|  | **† Daily Value (DV) not established.** | | |  |  |

**Supplementary Figure 1.** Nutritional information and ingredients for AG1^®^. ^††^Measured using viability digital PCR (dPCR), a method that quantifies intact and metabolically active probiotic cells.
