# Supplementary material for: Effect of AG1® supplementation on nutritional adequacy and gut microbial composition in trained adults
Source: Front Nutr. 2026 Mar 31;13:1783951. doi: 10.3389/fnut.2026.1783951 (PMC13077853; doi:10.3389/fnut.2026.1783951)
Supplement: Supplementary file 1 [file Supplementary_file_1.zip › Supplementary Figure 2.DOCX]

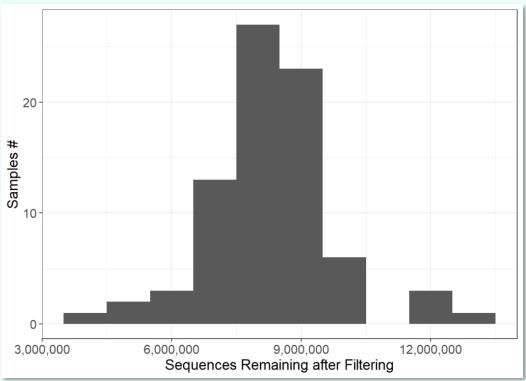


**Supplementary Figure 2.** A histogram representing the distribution of sequence counts. A total of 79 samples are represented. The range of sequences ranged from 4,473,000 to 12,642,550, with a total of 651,598,172 sequences and an average of 8.24 million sequences per sample after quality control.
