# Supplementary material for: Effect of AG1® supplementation on nutritional adequacy and gut microbial composition in trained adults
Source: Front Nutr. 2026 Mar 31;13:1783951. doi: 10.3389/fnut.2026.1783951 (PMC13077853; doi:10.3389/fnut.2026.1783951)
Supplement: Supplementary file 1 [file Supplementary_file_1.zip › Supplementary Figure 3.DOCX]

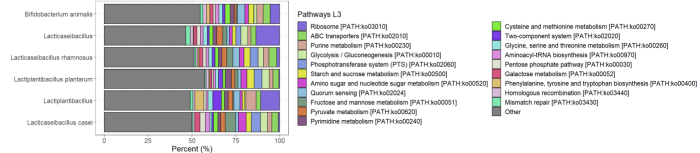


**Supplementary Figure 3.** A bar plot illustrating the functional pathway distribution of KEGG orthologs identified within the six biomarker taxa that was shown to be differentially enriched by AG1^®^ treatment. Prominent pathways include ribosome activity, ABC transporters, carbohydrate metabolism (e.g. glycolysis, starch and sucrose metabolism), amino sugar metabolism, and quorum sensing. These functional annotations indicate the probiotic taxa's role in supporting microbial growth, nutrient transport and utilization, and community regulation.
