# Supplementary material for: Effect of AG1® supplementation on nutritional adequacy and gut microbial composition in trained adults
Source: Front Nutr. 2026 Mar 31;13:1783951. doi: 10.3389/fnut.2026.1783951 (PMC13077853; doi:10.3389/fnut.2026.1783951)
Supplement: Supplementary file 1 [file Supplementary_file_1.zip › Supplementary Figure 4.DOCX]

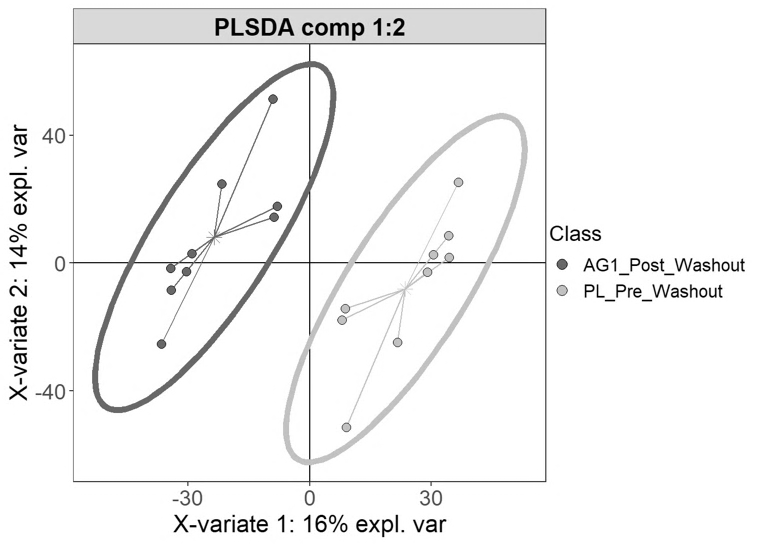


**Supplementary Figure 4.** Partial Least Squared Discriminant Analysis (PLS-DA) based on KEGG Ortholog relative abundance yields distinct clustering between Pre-Washout (Light) and Post-Washout (Dark) samples.
