# Supplementary material for: Effect of AG1® supplementation on nutritional adequacy and gut microbial composition in trained adults
Source: Front Nutr. 2026 Mar 31;13:1783951. doi: 10.3389/fnut.2026.1783951 (PMC13077853; doi:10.3389/fnut.2026.1783951)
Supplement: Supplementary file 1 [file Supplementary_file_1.zip › Supplementary Table 1.DOCX]

**Supplementary Table 1.** Top 10 KEGG Pathways associated with enriched genes in the AG1^®^ treatment group, showing the number of enriched genes mapped to each pathway.

| **KEGG Pathway** | **Enriched Genes Mapped Count** |
| --- | --- |
| ABC Transporters [PATH:KO02010] | 16 |
| Ribosome [PATH:KO03010] | 14 |
| Quorum Sensing [PATH:KO02024] | 13 |
| Starch and Sucrose Metabolism [PATH:KO00500] | 13 |
| Purine Metabolism [PATH:KO00230] | 12 |
| Cysteine and Methionine Metabolism [PATH:KO00270] | 9 |
| Peptidoglycan Biosynthesis [PATH:KO00550] | 9 |
| Glycine, Serine and Threonine Metabolism [PATH:KO00260] | 7 |
| Glycolysis / Gluconeogenesis [PATH:KO00010] | 7 |
| Pyrimidine Metabolism [PATH:KO00240] | 7 |
