# Supplementary material for: Effect of AG1® supplementation on nutritional adequacy and gut microbial composition in trained adults
Source: Front Nutr. 2026 Mar 31;13:1783951. doi: 10.3389/fnut.2026.1783951 (PMC13077853; doi:10.3389/fnut.2026.1783951)
Supplement: Supplementary file 1 [file Supplementary_file_1.zip › Supplementary Table 2.DOCX]

**Supplementary Table 2.** Significant KEGG orthologs identified by Linear Discriminant Analysis Effect Size (LEfSe) between Pre-Washout and Post-Washout groups.

| **Kegg ID/Name** | **Taxa** | **Group** | **LDA** | **P-value** |
| --- | --- | --- | --- | --- |
| K10112: ABC transporters - multiple sugar transport system ATP binding protein | *Blautia obeum* | PostAG1 - PrevWash | 0.572 | 0.001 |
| K02906: Ribosome - large subunit ribosomal protein L3 | *Blautia obeum* | PostAG1 - PrevWash | 0.539 | 0.000 |
| K01409: O-sialoglycoprotein endopeptidase | *Bifidobacterium animalis* | PostAG1 - PrevWash | 0.395 | 0.001 |
| K02520: translation initiation factor 3 (IF3) | *Eubacteriales* | PostAG1 - PrevWash | 0.382 | 0.001 |
| K01200: pullulanase | *Bifidobacterium animalis* | PostAG1 - PrevWash | 0.374 | 0.000 |
| K02864: Ribosome - large subunit ribosomal protein L10 | *Lachnospiraceae* | PostAG1 - PrevWash | 0.302 | 0.001 |
| K01809: mannose-6-phosphate isomerase (EC 5.3.1.8) | *Bifidobacterium animalis* | PostAG1 - PrevWash | 0.284 | 0.000 |
| K03791: magnesium chelatase family protein | *Bifidobacterium animalis* | PostAG1 - PrevWash | 0.283 | 0.001 |
