# Supplementary material for: Effect of AG1® supplementation on nutritional adequacy and gut microbial composition in trained adults
Source: Front Nutr. 2026 Mar 31;13:1783951. doi: 10.3389/fnut.2026.1783951 (PMC13077853; doi:10.3389/fnut.2026.1783951)
Supplement: Supplementary file 1 [file Supplementary_file_1.zip › Supplementary Table 4.DOCX]

**Supplementary Table 4.** Significantly altered metabolites which carry negative charges when ionized. Pre and Post values only pertain to AG1^®^ treated individuals to minimize metabolite changes irrelevant to the treatment effect. All units are arbitrary intensity units since untargeted metabolomics was conducted. P values are derived from paired T tests.

| **Metabolite Name** | **Average Pre Value** | **Average Post Value** | **P Value** | **Potential Biochemical Meaning** |
| --- | --- | --- | --- | --- |
| Deoxyribose | 50.58782182 | 31.81999702 | P = 0.021 | Reduced breakdown of nucleic acids |
| Mevalonic acid | 17.65888814 | 53.86021916 | P = 0.012 | Increased isoprenoid synthesis |
| 2-Indolecarboxylic acid | 68.31668833 | 34.88135842 | P = 0.036 | Altered tryptophan metabolism |
| Rhamnulose | 173.4785949 | 92.10274923 | P = 0.036 | Change in microbiome functionality |
| 3-Methylxanthine | 86.99694081 | 35.72386076 | P = 0.035 | Reduced caffeine catabolism |
| Xanthurenic acid | 47.85740935 | 21.04201564 | P = 0.048 | Altered tryptophan metabolism |
| 4-Acetyl-2(3H)-benzoxazolone | 394.4017438 | 138.6758892 | P = 0.02 | Change in microbiome functionality |
| 12-hydroxyheptadecanoic acid | 11.33432916 | 58.40330343 | P = 0.033 | Increased arachidonic acid pathway metabolism |
| 2,5,8,11,14-Pentaoxahexadecan-16-oic acid | 108.6592796 | 42.20542261 | P = 0.028 | Reduced exposure to PEG-related substances |
| 9,10-dihydroxyoctadecanoate | 501.2366346 | 984.4965389 | P = 0.013 | Increased metabolism of oleic acid |
| 12-Ketodeoxycholic acid | 2335.008611 | 1214.021534 | P = 0.024 | Altered bile acid metabolism |
| 27-Nor-5b-cholestane-3a,7a,12a,24,25-pentol | 159.5738467 | 241.0731324 | P = 0.012 | Increased bile alcohol synthesis |
| Medicagenic acid | 79.95325294 | 213.7725236 | P = 0.014 | Increased metabolism of plant material |
